# Morin ameliorates myocardial injury in diabetic rats via modulation of inflammatory pathways

**DOI:** 10.1186/s42826-024-00190-x

**Published:** 2024-02-09

**Authors:** Vipin Kumar Verma, Salma Malik, Ekta Mutneja, Anil Kumar Sahu, Vaishali Prajapati, Prashant Mishra, Jagriti Bhatia, Dharamveer Singh Arya

**Affiliations:** 1 Cardiovascular Research Laboratory, Department of Pharmacology, All India Institute of Medical Sciences, New Delhi, 110029 India; 2 Department of Pharmacology, Armed Force Medical College, Pune, Maharastra 411040 India

**Keywords:** Isoproterenol, Diabetes, Myocardial necrosis, Molecular signaling pathway

## Abstract

**Background:**

High blood glucose levels in diabetes lead to vascular inflammation which accelerates atherosclerosis. Herein, Morin was orally administered in male Wistar rats, at the dose of 40 mg/kg for 28 days, and on the 27th and 28th day, ISO was administered to designate groups at the dose of 85 mg/kg s.c., to induce myocardial infarction.

**Results:**

Free radical generation, including ROS, in diabetes following ISO administration, leads to the activation of both intrinsic and extrinsic pathways of apoptosis. Morin significantly (*p* ≤ 0.05) reduced oxidative stress (GSH, MDA, SOD), cardiac injury markers (CK-MB, LDH), inflammation (TNF, IL-6), and apoptosis (Bax, BCl_2_, Caspase-3). In addition, it also reduced insulin and blood glucose levels. Akt/eNOS, Nrf2/HO-1, MAPK signaling pathways, and Insulin signal transduction pathways were positively modulated by morin pre-treatment.

**Conclusions:**

Morin attenuated oxidative stress and inflammation and also modified the activity of various molecular pathways to mitigate cardiomyocyte damage during ISO-induced MI in diabetic rats.

**Supplementary Information:**

The online version contains supplementary material available at 10.1186/s42826-024-00190-x.

## Background

Diabetes is a group of metabolic disorders having increased blood sugar due to a decrease in insulin secretion and action, or both. Worldwide, 10.5% prevalence of diabetes mellitus (DM) aged between 20 and 79 years was estimated by International Diabetes Federation (IDF) in the year 2021 which is predicted to be rise by 11.3% and 12.2% by the year 2030 and 2045 respectively [1] Hyperglycemia is a main risk factor for the development of cardiovascular diseases (CVDs) such as myocardial infarction (MI), atrial fibrillation (AF), heart failure (HF), and cardiomyopathy. Also, the mortality rate due to CVDs is 3 to 4 times higher in DM patients than non-DM population [2].

Several studies have shown that hyperglycemia can cause the production of advanced glycation end (AGE) products, interact with its receptor (RAGE), and lead to oxidative stress, apoptosis, and inflammation [3]. To protect cardiac cells from oxidative stress, the nuclear factor erythroid 2- related factor/heme oxygenase-1 (Nrf2/HO-1) pathway is activated. Normally, Nrf2 resides in the cytoplasm with an inhibitory protein called keap. With increased oxidative stress, Nrf2 detaches from keap, translocates to the nucleus, and activates various antioxidants such as HO-1, superoxide dismutase (SOD), catalase, glutathione transferase, etc., which reduces the deleterious effect of reactive oxygen species [4]. Various other pathways are also activated during myocardial injury, among which phosphoinositide 3-kinase (PI3K)/Akt/glycogen synthase kinase (GSK)-3β pathway regulates numerous cellular functions such as cell proliferation, apoptosis, and cell survival. Also, previous studies have shown protection against heart failure in diabetes via the regulation of Nrf2/HO-1 and PI3K/Akt pathways [5, 6]. Furthermore, it also activates the mitogen-activated protein kinase (MAPK) pathway, resulting in inflammation and apoptosis in the myocardium [7]. Thus, there is a cross-talk between the signaling pathways which on activation can either protect or produce a deleterious effect in the myocardium.

The rising prevalence of diabetes-related cardiac dysfunction requires a focus on finding a new drug moiety that can prevent the deteriorating function of the heart during high metabolic demands. Morin (3,5,7,2’,4’-pentahydroxy flavone) a flavonoid present in *Prunus dulcis*, *Cudrania tricuspidata*, *Morus alba* and *Psidium guajava*. Morin acts through its antioxidant and anti-inflammatory properties and has been found effective against several disease pathologies such as liver injury, cerebral ischemia reperfusion, cancer, cardiovascular, and renal complications [8–12]. Previous studies have documented the cardioprotective role of morin against myocardial injury [13]. Paoli and his co-workers found insulin mimicking effect of morin [14]. In addition, Razavi and his colleagues showed the antidiabetic potential of morin through inhibition of miR-29a [15]. However, the potential effect of morin has not been assessed on cardiac dysfunction due to DM. Hence, in the present study, we investigated the effect of morin on myocardial injury in streptozotocin-induced diabetic rats.

## Methods

### Drugs and chemicals

Morin, isoproterenol and streptozotocin were procured from Sigma-Aldrich, USA. Enzyme linked immunosorbent assay kits for TNF (Tumor necrosis factor), Caspase-1, IL-6 (Interleukin-6), IL-10, NLRP-3 (NACHT, LPR and PYD Domain-containing Protein-3) were purchased from CusaBio Technology LLC, USA. However, ELISA kit for AGE estimation was purchased from Korain Biotech Co. Ltd., China. Creatine Kinase-MB (CK-MB) and Lactate dehydrogenase (LDH) were obtained from Elab Sciences, Texas, USA. Insulin kit was purchased from RayBiotech, GA, USA. Primary antibodies, i.e., Akt, P-Akt, endothelial nitric oxide synthase (eNOS), GSK-3β, IKK-β, P- IKK-β, NF-κBp65, P-NF-κBp65, extracellular regulated kinase 1/2 (ERK1/2), P-ERK1/2, c-Jun N-terminal kinase (JNK), P-JNK, p38, Phospho-p38, P53, Bax, Bcl-2, caspase-3, Nrf-2, HO-1, cytochrome-C, FABP and HMGB1 were purchased from Abcam, California, USA. However, antibodies for AMPK, P-AMPK, HSP-70, HSP-27, HSP-20, Cyclin D1 and RAGE were purchased from Santa Cruz Biotechnology, Inc., California, USA. Antibody for PARP was procured from G-Biosciences, St. Louis, USA, while the GAPDH antibody (raised in mice) was procured from AbbKine, China. HRP-conjugated secondary antibodies (Goat anti-Rabbit and Goat anti-Mouse) were procured from Thermo Fisher Scientific, USA, while the fluorescent (PE) labelled antibody was purchased from Abcam, California, USA. All chemicals used in the study were of Molecular Biology grade.

### Experimental animals and conditions

Male Wistar albino rats aged 10–12 weeks (150–200 g) were procured from the institutional central animal facility at AIIMS-New Delhi, after ethical approval (Approval letter number: 03/IAEC-1/2017 dated October 17, 2017) from the Institutional Animal Ethics Committee, AIIMS-New Delhi (Registration Number: 10/GO/ReBi/S/99/CPCSEA). The research work was conducted as per Indian National Science Academy (INSA-CPCSEA) guidelines (following ARRIVE guidelines of animal research) for the care and use of animals in scientific research. and were acclimatized in the departmental animal house in polypropylene cages of 40 × 25x15 cm size. The temperature and relative humidity of the premises was maintained at 25 ± 2 °C and 60 ± 5%, respectively. The light and dark cycle was maintained for 12:12 h and animals were fed with chow diet and water ad libitum.

### Streptozotocin-induced type-1 diabetes in rats

For induction of diabetes, all rats were subjected to overnight fasting followed by administration of streptozotocin (STZ), i.e., 70 mg/kg prepared in ice-chilled 0.1 M Citrate buffer (pH 4.5); intraperitoneally. On 3rd and 7th day after STZ administration and subsequently weekly during experimentation, fasting blood glucose levels were assessed using a OneTouch Glucometer (SD Biosensor, India). Rats with fasting blood glucose levels > 400 mg/dl were considered type-1 diabetic and included in the study.

### Study design

**Fig. 1:**
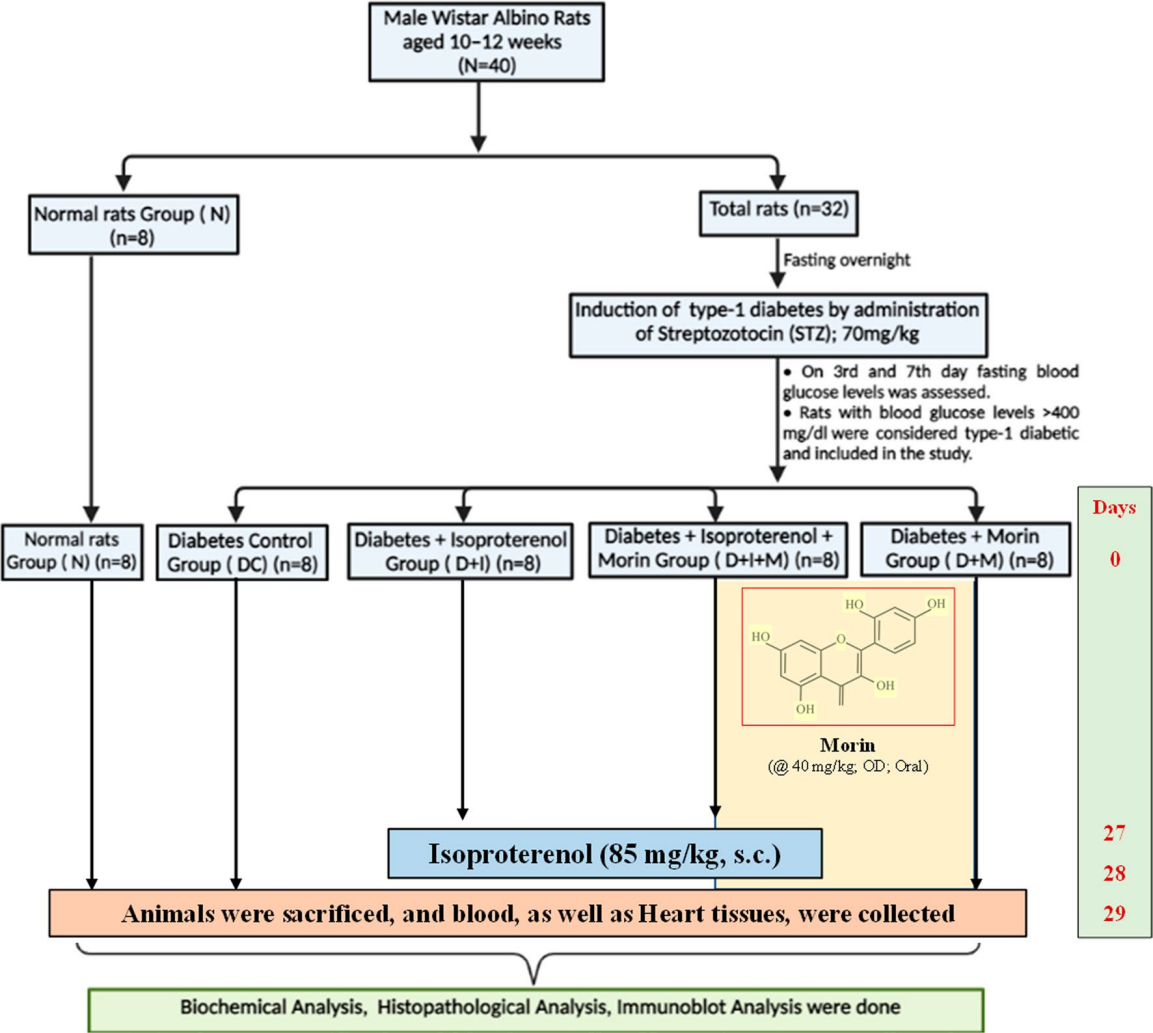
Schematic representation of study protocol

The flowchart of the study design is shown in Fig. 1. A total of 40 animals were randomly divided into five groups (n = 8 in each group). The grouping of animals was as follows; Group N: Normal; Group DC: Diabetes control; Group D + I: Diabetes + Isoproterenol (ISO); Group D + I + M: Diabetes + ISO + Morin 40 mg/kg; Group D + M: Diabetes + Morin 40 mg/kg. The test drug (Morin) was dissolved in normal saline (0.9% NaCl) and administered at the dose of 40 mg/kg, orally to the rats of groups D + I + M & D + M for 28 days [13]. In the N, DC, and D + I groups, normal saline (1 ml/kg) was administered. On days 27th and 28th, animals of groups D + I and D + I + M were injected with isoproterenol at 85 mg/kg subcutaneously at an interval of 24 h. On 29th day, animals were anesthetized with pentobarbitone sodium at 60 mg/kg, intraperitoneally.

Tracheostomy was performed in all rats by ventral midline incision in the neck region. The rats were ventilated with room air from a respirator (RWD, China). The right carotid artery was cannulated with a polypropylene tube connected to the pressure transducer of the Biopac system (Software BSL 4.0 MP36), for maintaining hemodynamics. Thereafter, thoracotomy was performed between 5 and 6th intercostal space using cautery. 2 ml of blood was withdrawn through cardiac puncture from the left ventricular heart. Animals were euthanized with an overdose of anesthesia to excise heart tissue.

Isolated serum and a part of the heart tissue from each animal were snap-chilled using liquid nitrogen and stored at -86 °C for further analysis of biochemical and molecular parameters. However, a part of the heart tissue was stored in 10% neutral formaldehyde for histopathological evaluation.

### Biochemical analysis

Heart tissue was removed from − 86 °C, weighed, and pulverized to make 10% homogenate in ice-chilled phosphate buffer (0.1 M, pH 7.4). The homogenate was stored in 2 aliquots. The first aliquot was used for the estimation of malondialdehyde (MDA) and reduced glutathione (GSH) as per the protocol given in Ohkawa et al. and Moron et al. respectively [16, 17]. The second part of the aliquot was centrifuged at 6500 rpm; supernatant was separated and used for the estimation of protein concentration using Bradford reagent [18] and enzymatic activity of superoxide dismutase (SOD) using the standardized protocol of Marklund & Marklund [19].

### Histopathological analysis

The tissue stored in formalin was embedded in paraffin wax to make blocks following the standardized dehydration and xylene saturation process. From these blocks, thin Sects. (5 µm thick) were cut using a microtome and taken on egg albumin precoated slides. The sections were deparaffinized and stained with hematoxylin and eosin (H&E) for histopathology. The slides were visualized under a light microscope (Dewinter technologies, Italy) by a pathologist blinded to the study groups. Structural damage was recorded from the slides and images were captured.

### Estimation of serum markers

Serum separated from the blood samples was used for the estimation of cardiac injury markers (CK-MB, LDH), Insulin, and inflammatory markers (TNF, IL-6, IL-1β, NLRP3 and caspase-1) using ELISA kits according to the manufacturer’s protocol.

### Western blot analysis

Stored heart tissue was homogenized in RIPA buffer along with a protease inhibitor (MS-SAFE, Sigma-Aldrich, USA). Homogenate was then centrifuged at 10,000 rpm for 15 min and the protein concentration in the supernatant was estimated using Bradford’s assay. A total of 40 μg of proteins were separated on 12% denatured resolving gel through polyacrylamide gel electrophoresis (PAGE) in reducing condition and transferred to a nitrocellulose membrane (0.2 µm; Bio-rad, USA). Thereafter, the membrane was blocked with BSA (3%) for 2 h at RT. The membrane was incubated overnight with the respective primary antibodies along with the GAPDH antibody, followed by the particular secondary antibodies in a specific combination for 2 h at RT. After that, the bands having HRP-conjugated antibody were detected by the enhanced Chemiluminescence (ECL) method using ECL kit (Clarity western enhanced luminescence kit, Bio-rad, USA) while the fluorescence (PE)-labelled antibody was detected using MultiFluor Red channel with red epi light and Far Red (710 nm) filter under gel documentation system, FluorChem M, Protein Simple, California, USA.

### Statistical analysis

Data from all groups (n = 6 animal of each group) were analyzed using one-way ANOVA followed by Bonferroni post hoc test using Sigma Plot 12.0 software. Data were expressed as mean ± SEM and *P*-value < 0.05 was considered as statistically significant.

## Results

### Effect on body weight, blood glucose, and serum insulin levels

**Fig. 2:**
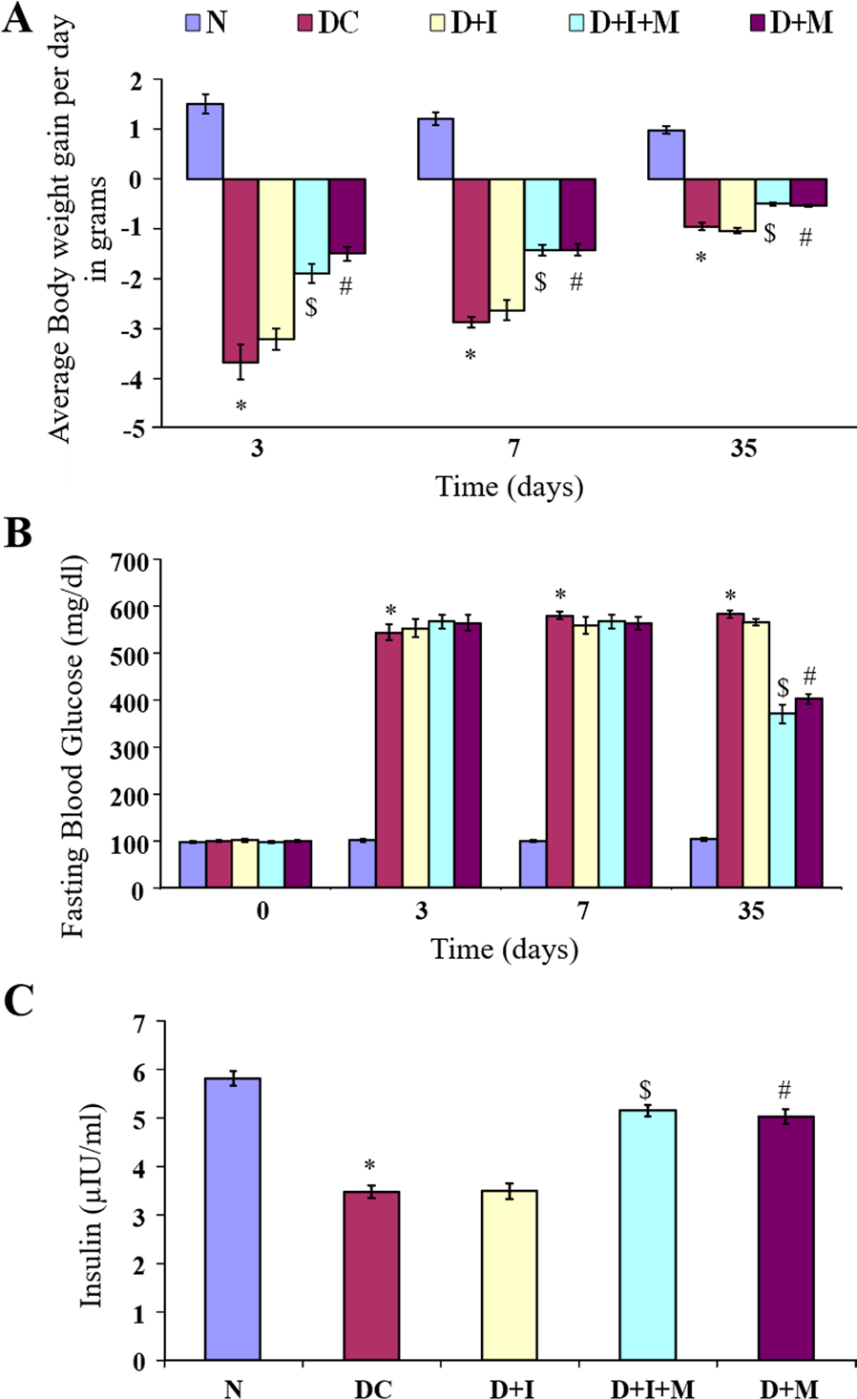
Effect of morin on animal weight, blood glucose and Insulin concentration. **A**. average weight gain per day in grams [(Present weight – previous weight)/ Number of days)]; **B**. Fasting blood glucose levels in mg/dl, and **C**. serum insulin levels in µIU/ml. Statistical analysis was performed using one-way ANOVA followed by Bonferroni multiple comparison tests. All values are expressed as mean ± SEM (n = 6). Error bars with different superscripts are shown as significantly different (*P* < 0.05) where “*” indicates statistical difference between N vs DC; “#” specify the difference between DC vs D + I and D + M; however “$” shows statistical difference in between D + I vs D + I + M). N: Normal; DC: Diabetes control; D + I: Diabetes + Isoproterenol (ISO); D + I + M: Diabetes + ISO + Morin 40 mg/kg; D + M: Diabetes + Morin 40 mg/kg

Average body weight gain and blood glucose levels of all rats were measured on the 3rd, 7th, and 35th day of the experiment. There was a significant reduction in body weight and an increase in blood glucose levels were observed in all diabetic rats in comparison to the normal group throughout the experimental period of 35 days (Fig. 2A and B). Moreover, the average decrease in body weight and blood glucose levels show a significant decrease in groups with morin treatment (D + M and D + I + M) in comparison with D + I.

In diabetic control rats, there was a significant decrease in insulin levels in comparison to the normal group. Treatment with morin significantly improved serum insulin levels when compared to DC and D + I group rats (*P* < 0.05) (Fig. 2C).

## Determination of oxidant-antioxidant levels and cardiac injury markers

**Fig. 3:**
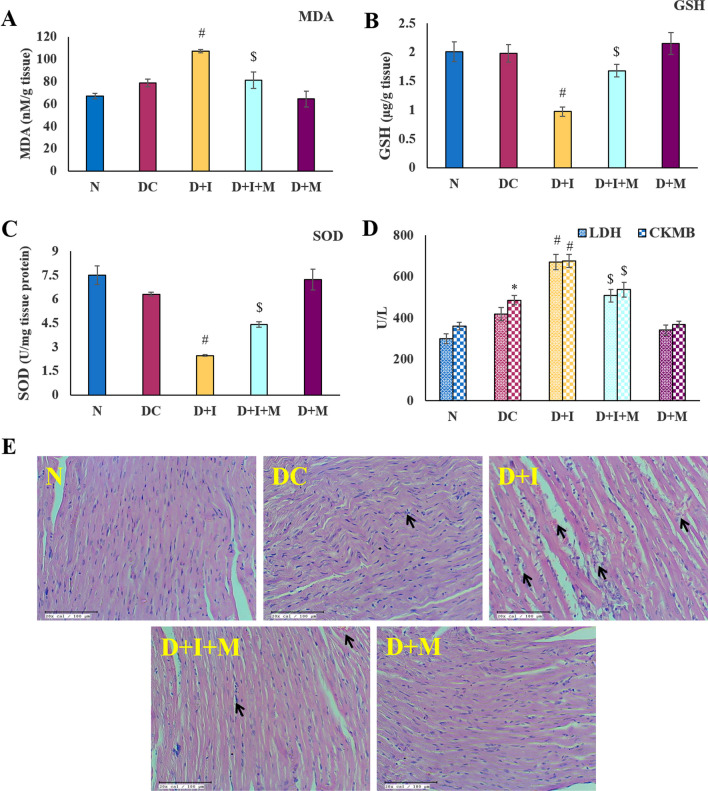
Effect of morin on biochemical parameters, cardiac-injury markers, and Pathological changes **A**.MDA (Malonaldehyde) levels in nM per gram heart tissue weight. **B**. GSK (Reduced glutathione) levels in µg present per gram of rat heart tissue weight. **C**. SOD (superoxide dismutase) levels in the Unit of SOD present per gram of heart tissue weight. **D**. LDH (Lactate dehydrogenase) and CK-MB (Creatine Kinase-MB) levels in rat serum in Units present per liter of serum. Statistical analysis was performed using one-way ANOVA followed by Bonferroni multiple comparison tests. All values are expressed as mean ± SEM (n = 6). Error bars with different superscripts are shown as significantly different (*P* < 0.05) where “*” indicates statistical difference between N vs DC; “#” specify the difference between DC vs D + I and D + M; however “$” shows statistical difference in between D + I vs D + I + M). N: Normal; DC: Diabetes control; D + I: Diabetes + Isoproterenol (ISO); D + I + M: Diabetes + ISO + Morin 40 mg/kg; D + M: Diabetes + Morin 40 mg/kg. E. Effect of morin on histopathological changes in rat models of diabetes and myocardial injury (200x; scale bar 100 μm; n = 3). Arrow represents the myocardial damage as infiltration of immune cells, myocardial membrane damage, and necrosis. a. Normal; b. Diabetes control; c. Diabetes + Isoproterenol (ISO); d. Diabetes + ISO + Morin 40 mg/kg; e. Diabetes + Morin 40 mg/kg

In D + I group, there was a significant increase in the level of MDA (a marker of lipid peroxidation) and a decrease in the levels of antioxidants (GSH and SOD) in comparison to the DC group (*P* < 0.05). In addition, there was an increased level of cardiac injury markers (CK-MB and LDH) in DC and D + I rats in comparison to normal rats (*P* < 0.05). This indicates the production of free radicals after the administration of STZ to the rats, which was further aggravated by ISO administration to the diabetic rats. However, treatment with morin in diabetic rats significantly restored the oxidant-antioxidant status (MDA, GSH, and SOD) and decreased the levels of cardiac injury markers when compared to DC and D + I rats (*P* < 0.05) even after ISO administration as shown in Fig. 3A–D.

## Determination of myocardial architecture

In the pathological evaluation, normal control group rats showed well-preserved myocardium with no evidence of edema and inflammation. However, in DC group rats, there was a mild infiltration of inflammatory cells which showed waviness in myofibrils along with endothelial swelling. Furthermore, in D + I group rats, there was marked inflammation, myonecrosis, edema, and myocardial damage (pointed out using black arrows). Morin treatment for 28 days in D + I + M group rats showed considerable improvement with a decrease in inflammatory cells, reduced inflammation, less necrosis, and mild cardiomyocyte edema when compared with the D + I group. However, on comparing D + M to the DC group significantly improved myocardial texture with very less inflammation (Fig. 3E).

## Effect on apoptosis

To confirm the presence of apoptosis, the level of apoptotic markers (Bax, Bcl-2, caspase-3, cytochrome-c, PARP, and p53) were measured by western blot analysis. There were increased levels of Bax, caspase-3 and decreased levels of Bcl-2, cytochrome c, PARP, and p53 in DC and D + I groups in comparison to normal group rats (*P* < 0.05). Morin combination groups showed a reduction in Bax dimer, cytochrome-c, and P53 along with PARP (D + M) and Bcl-2 (D + I + M) in comparison with non-morin groups except normal group. The levels of caspase-3 were obtained higher in morin treatment groups with PARP (D + I + M) for a period of 35 days.

**Fig. 4:**
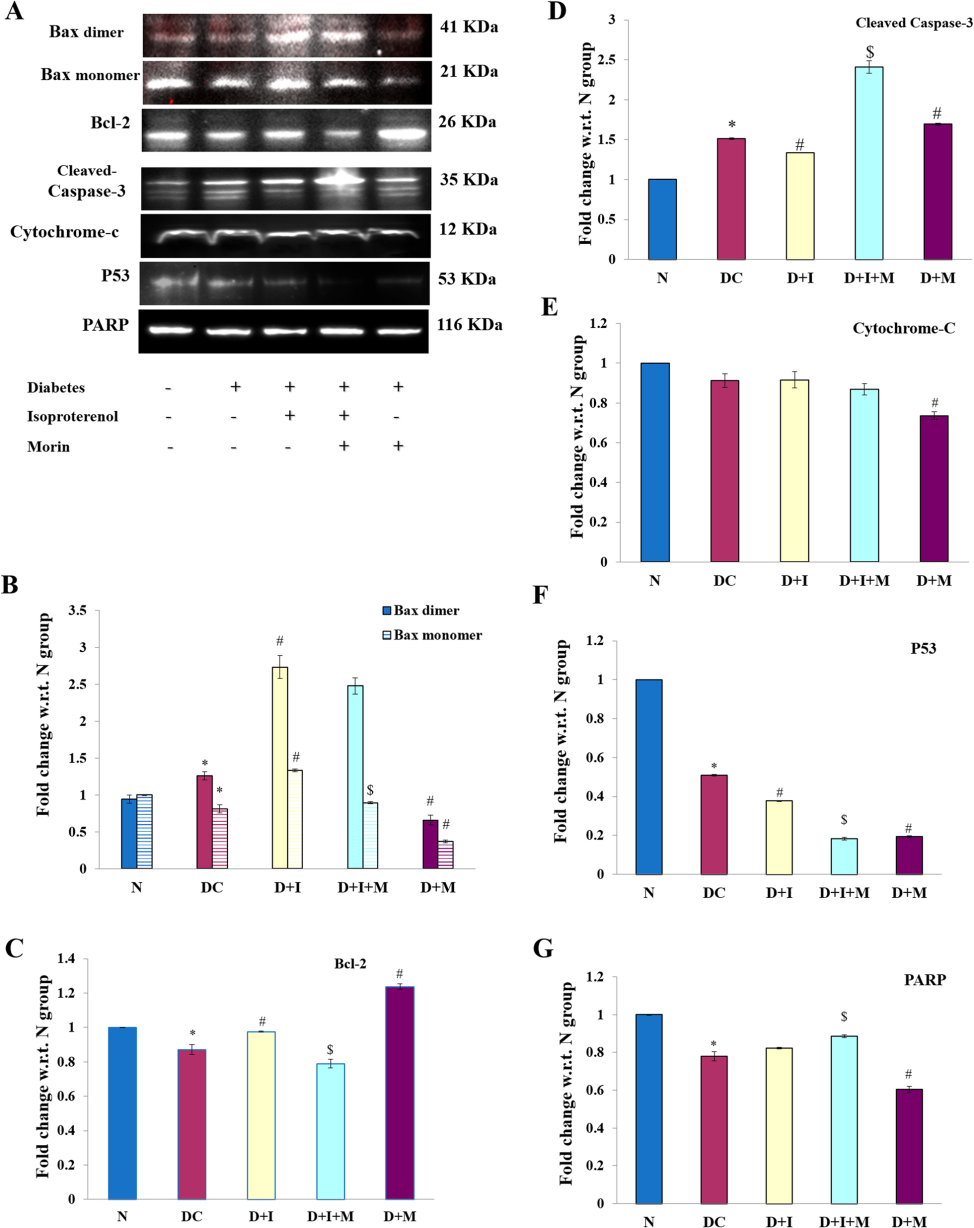
Effect of morin on apoptotic pathway proteins **A**.Representative western blotting images showing expression of apoptotic pathway proteins. Graphical representation of relative densitometric quantification w.r.t. respective loading control and normalized with Normal group. **B**. Bax dimer and monomer. **C**. Bcl-2. **D**. Cleaved-Caspase-3. E. Cytochrome-C. F. p53. G. PARP in rat heart tissue. Statistical analysis was performed using one-way ANOVA followed by Bonferroni multiple comparison tests (n = 3). Error bars with different superscripts are shown as significantly different (*P *< 0.05) where “*” indicates statistical difference between N vs DC; “#” specify the difference between DC vs D + I and D + M; however “$” shows statistical difference in between D + I vs D + I + M). N: Normal; DC: Diabetes control; D + I: Diabetes + Isoproterenol (ISO); D + I + M: Diabetes + ISO + Morin 40 mg/kg; D + M: Diabetes + Morin 40 mg/kg

Treatment with morin positively modulated the level of apoptotic markers and thus attenuated apoptosis in diabetic rats (*P* < 0.05) (Fig. 4A–F).

## Effect on MAPK and inflammatory pathway

**Fig. 5:**
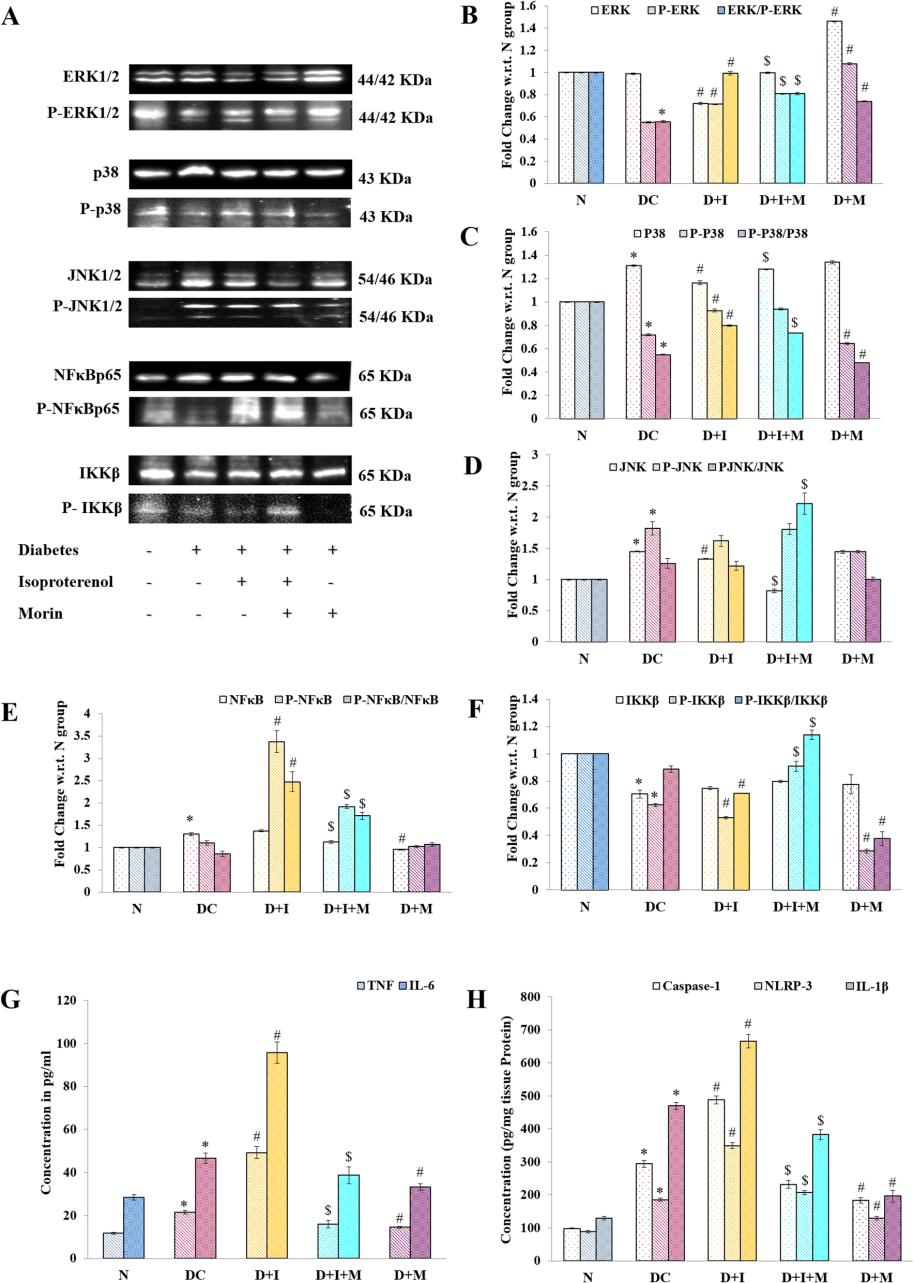
Effect of Morin on MAPK and inflammatory pathway proteins **A**.Representative western blots and their densitometric quantification w.r.t. respective loading control. Bars represent the relative intensity of respective protein normalized with Normal group. a. MAPK-ERK/ P-ERK. b. P38/ P-P38. c. JNK/ P-JNK. d. NF-κBp65/ P- NF-κBp65. e. IKKβ/ P-IKKβ. **B**. Concentration of inflammatory cytokines TNF and IL-6 estimated through ELISA following manufacturer’s protocol. **C**. Concentration of inflammasome proteins Caspase-1, NLRP-3 and IL-1β proteins. Statistical analysis was performed using one-way ANOVA followed by Bonferroni multiple comparison tests (n = 3). Error bars with different superscripts are shown as significantly different (*P* < 0.05) where “*” indicates statistical difference between N vs DC; “#” specify the difference between DC vs D + I and D + M; however “$” shows statistical difference in between D + I vs D + I + M). N: Normal; DC: Diabetes control; D + I: Diabetes + Isoproterenol (ISO); D + I + M: Diabetes + ISO + Morin 40 mg/kg; D + M: Diabetes + Morin 40 mg/kg

In comparison to normal rats, there were decreased levels of ratio of ERK1/2 / P-ERK1/2, P38 / p-P38, while an increased levels of JNK / P-JNK, in DC group was observed. However, significantly increased levels of P38/p-P38 was observed in D + I group w.r.t. DC group. While on morin administration, significantly decreased levels of p-P38 were observed hence the ratio also decreases. Increased levels of P38 and JNK further lead to the activation of the inflammatory cascade in the myocardium. Thus, there was an increased level of NF-κB and inflammatory cytokines (TNF and IL-6) and decreased levels of IKK-β was observed in the DC and D + I groups. In contrast, treatment with morin significantly decreases to normalized the level of the MAPK signaling pathway proteins and reduced the level of inflammatory markers and inflammasome proteins (caspase-1, NLRP-3, and IL-1β) (*P* < 0.05) (Fig. 5A–C).

## Effects on insulin signaling pathways proteins, FABP, HMGB1, and HSP expressions

**Fig. 6:**
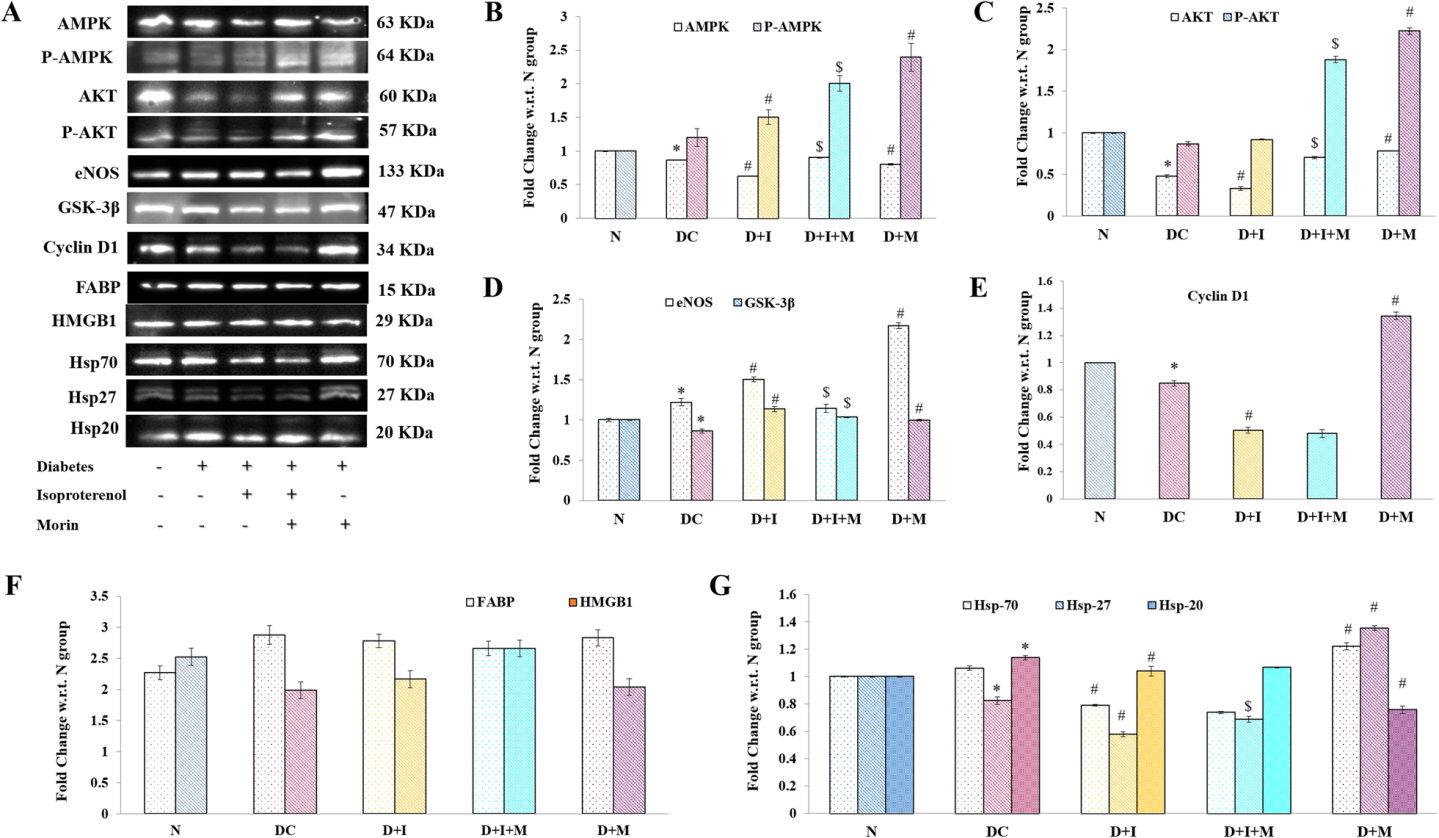
Effect of morin on Insulin signaling pathway proteins, FABP, HMGB and HSP expressions. **A**. Representative western blot images of different study group animals (n = 3). The bar graph represents the relative band intensity w.r.t. respective loading control and normalized with Normal group. AMPK/ P-AMPK **B**; AKT/ P-AKT **C**; eNOS/ GSK-3β **D**; Cyclin D1 **E**; FABP and HMGB-1 **F**; Heat shock proteins (70/ 27/ 20) **G**. Statistical analysis was performed using one-way ANOVA followed by Bonferroni multiple comparison tests (n = 3). Error bars with different superscripts are shown as significantly different (*P* < 0.05) where “*” indicates statistical difference between N vs DC; “#” specify the difference between DC vs D + I and D + M; however “$” shows statistical difference in between D + I vs D + I + M). N: Normal; DC: Diabetes control; D + I: Diabetes + Isoproterenol (ISO); D + I + M: Diabetes + ISO + Morin 40 mg/kg; D + M: Diabetes + Morin 40 mg/kg

There was increased expression of p-AMPK, and p-AKT were observed in morin treatment groups with diabetes and also after administration of ISO (Fig. 6B, C). In comparison to DC group the expression of eNOS, GSK-3β, and Cyclin-D1 were also upregulated on morin administration in D + M group rats (Fig. 6D–E). However, no significant changes were observed in FABP and HMGB1 protein expression in between the groups. Furthermore, there was significantly increased expression of Hsp70, Hsp27, were also observed in the D + M group in comparison to DC while the levels were significantly less in D + I group (*P* < 0.05) (Fig. 6G). While no significant changes were observed in Hsp-70 and Hsp-20 considerably high levels of Hsp-27 (*P* < 0.05).

## Effect on Nrf2/HO-I and AGE-RAGE

**Fig. 7:**
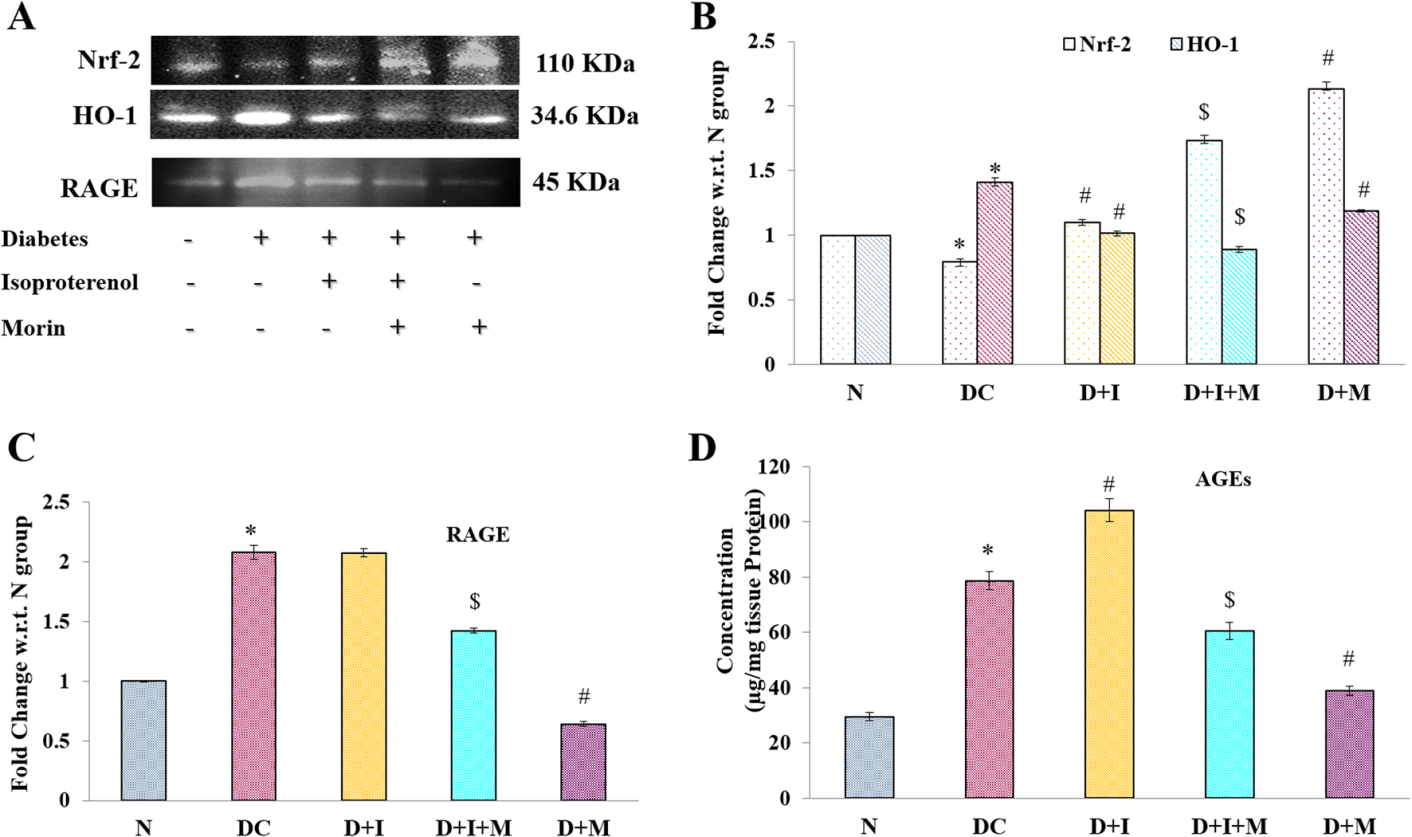
Effect of morin on Nrf-2/HO-1 and AGE-RAGE **A**. Representative western blot images of Nrf-2/ HO-1 and RAGE of study groups. The bar graphs showing relative band intensity w.r.t. respective loading control normalized with Normal group for Nrf-2/ HO-1 **B** and RAGE **C**. The concentration of AGEs were measured from ELISA following manufacturer’s protocol and represented in ug of AGEs present per mg of heart tissues **D**. Statistical analysis was performed using one-way ANOVA followed by Bonferroni multiple comparison tests (n = 3). Error bars with different superscripts are shown as significantly different (*P* < 0.05) where “*” indicates statistical difference between N vs DC; “#” specify the difference between DC vs D + I and D + M; however “$” shows statistical difference in between D + I vs D + I + M). N: Normal; DC: Diabetes control; D + I: Diabetes + Isoproterenol (ISO); D + I + M: Diabetes + ISO + Morin 40 mg/kg; D + M: Diabetes + Morin 40 mg/kg

Researchers have demonstrated that morin increases the level of GSH, and SOD in the myocardium of diabetic rats. It is well known that these antioxidants are the downstream pathways of the Nrf2 pathway, thus assessing the expression of Nrf2/HO-1 by western blot analysis. There was significantly decreased expression of Nrf2 and increased expression of HO-1 was observed in the diabetic rats (DC), while on ISO administration the high levels of Nrf-2 and lower levels of HO-1 was observed in D + I group in comparison to DC.The treatment with morin significantly increases the levesl of Nrf-2 to minimize the oxidative damage (Fig. 7A, B). However, increased expression of AGEs and RAGE were also observed in DC gorup rats which were significantly attenuated with morin treatment (Fig. 7C, D).

## Discussion

Cardiovascular, cerebrovascular, diabetes, liver, and kidney diseases through antioxidant, antiapoptotic, and anti-inflammatory mechanisms. However, the effect of morin on myocardial injury in the diabetic condition is still unknown. In the present study, the effect of morin on ISO-induced myocardial injury in diabetic rats was evaluated. In the results, we found that morin reduced blood glucose and serum insulin levels, strengthened the antioxidant defense system, preserved myocardial architecture, and prevented the increase in inflammation and apoptosis in the myocardium. This could be due to the regulation of multiple signaling pathways, i.e. AGE-RAGE/Nrf2/HO-1, AMPK, and GSK-3β/Akt/eNOS.

Streptozotocin is an antibiotic that is widely used to produce diabetes in experimental animals. It enters the pancreatic β cells via the GLUT2 transporter. In β cells, it has a cytotoxic effect, causing DNA fragmentation, depletion of intracellular NAD + and ATP, and mitochondrial dysfunction, which ultimately leads to necrosis of pancreatic β cells. A decrease in the number of pancreatic β cells produces a relative or absolute decrease in insulin levels resulting in an increase in blood glucose levels [20]. A decrease in insulin level also leads to the breakdown of proteins, and fatty acids, as well as increased muscle wasting which reflects a decrease in body weight [21]. Similarly, in the present study, we found that an increase in hypo-insulinemia, hyperglycemia, and a decrease in body weight in diabetic group animals and morin treatment significantly reversed (but not completely) their levels.

It has been known that hyperglycemia activates the production of AGE, which then interact with RAGE and enhance the production of free radicals [3]. When diabetic rats were further subjected to isoproterenol, it led to massive production of free radicals and depletion of the antioxidant defense system. Therefore, free radicals further react with lipids, proteins, and DNA which causes cell membrane damage. Subsequently leads to lipid peroxidation and the release of cardiac injury markers (CK-MB & LDH) from the cell membrane. Previously, various studies have shown that natural antioxidants protect against myocardial injury in diabetic rats by inhibiting the AGE/RAGE pathway [22, 23]. With increased oxidative stress, the body activates the antioxidant system which prevents the generation of free radicals, lipid peroxidation, and depletion of antioxidants. Throughout our study, we discovered that the Nrf2 pathway was activated in order to defend against free radicals, as previously demonstrated by other researchers. On activation, Nrf2 binds to the antioxidant response elements (ARE) and increases the production of various antioxidant enzymes [4, 6]. In line with this, we extended the analysis and found higher levels of AGE/RAGE proteins, MDA (a marker of lipid peroxidation), cardiac injury markers, and decreased antioxidants (GSH and SOD) in the DC and D + I group rats. Alternatively, treatment with morin reduced the AGE/RAGE levels, lipid peroxidation, LDH, and CK-MB levels while increasing antioxidant status, which could be due to the activation of Nrf2 pathway. In consistent with our results, previous studies have demonstrated the antioxidant role of morin in various disease models [8, 10, 13, 24].

Furthermore, to explore the effect of morin on apoptosis in this model, various antiapoptotic and proapoptotic protein levels were measured. The intrinsic pathway of apoptosis involves the release of cytochrome c from mitochondria to the cytosol where it binds to Apaf-1 and causes activation of the caspase-dependent apoptotic pathway. Bcl-2 can inhibit apoptosis by preventing the release of cytochrome c from the mitochondrial membrane. Puma and Noxa (BH3- only proteins) are two members of Bcl-2 family which are involved in apoptosis and have been found to be stimulated by p53. Both intrinsic and extrinsic pathways of apoptosis terminate at the executioner phase of apoptosis. Caspase- 3, 7, and 9 are members of the executioner phase of apoptosis which further cleaves various cytokines such as PARP leading to biochemical and morphological changes in the apoptotic cells [25]. Previously, it has been found that Hsp 70 and 27 act at different steps of the intrinsic pathway of apoptosis and prevent apoptosis [26, 27]. In our study, rats in DC and D + I groups had lower levels of antiapoptotic proteins (Hsp 70, 27, and Bcl-2) and higher levels of proapoptotic markers (cytochrome c, Bax, caspase-3, PARP, and p53), which was reversed in the morin treatment group. In accordance with our findings, various other studies have shown the antiapoptotic potential of morin in different tissues/ organs [8, 28, 29]. Furthermore, it has been documented that Akt/GSK-3β from the insulin signaling pathway regulates apoptosis and promotes cell survival, while phosphorylation of Akt has been shown to reduce apoptosis [30]. In line with this, we found an increased level of Akt in the morin treatment, which might be attributed to decrease levels of proapoptotic proteins in the treatment group. Rizvi et al. have shown the protective effect of morin through the activation of the Akt/GSK-3β pathway [31].

It has been well-documented that sustained oxidative stress can lead to the activation of the MAPK pathway. MAPK pathway consists of three proteins: ERK, JNK, and P38. Activation of JNK and P38 has been shown to induce inflammation in the myocardium by stimulation of transcription factors such as NF-κB/IKKβ [32]. In the inactive form, NF-κB resides in the cytoplasm with the inhibitory protein IκB. In response to stressed conditions, there is phosphorylation of IκB by IKK which causes nuclear translocation of inflammatory cytokines, i.e., TNF and IL-6. HMGB1 has also been shown to increase the production of inflammatory cytokines by interacting with RAGE and toll-like receptors [33]. In this study, there was an increased level of MAPK, NF-κB/IKKβ, HMGBI, and inflammatory cytokines in the DC and D + I group rats, which were significantly reduced by morin treatment. Recent studies have shown that morin can act as an anti-inflammatory agent by inhibiting inflammatory pathways [8, 28].

Recently, the role of inflammasomes has been extensively studied in myocardial ischemic injury, Alzheimer’s disease, and diabetes [34]. NLRP3 inflammasome consists of NLRP3, cysteine protease pro-caspase-1, and the adaptor protein apoptosis-associated speck-like protein containing a caspase recruitment domain (ASC). In response to oxidative stress, NLRP3 forms a complex with ASC which results in caspase-1 activation. Activated caspase-1 induces the cleavage of IL-1β and IL-18 to their active form, which causes tissue inflammation. Activation of caspase-1 also leads to inflammation-related cell death known as pyroptosis [35]. Li et al. have shown that morin protects against Listeria monocytogenes-induced infection by inhibition of the inflammasome pathway [36]. In the present study, there was an increased level of inflammasomes (NLRP3, IL-1β, and caspase-1) in the DC and D + I group, and treatment with morin reduced levels of inflammasome proteins. All the representable western blots images and their respective GAPDH are present in Additional file 1. This study will give information on using morin or morin-rich food to reduce blood sugar, oxidative stress, inflammation, and cardiovascular complications in diabetic patients. Further clinical studies are also required to confirm this pre-clinical study.

## Conclusions

The present mechanistic study demonstrated that morin attenuated oxidative injury, apoptosis, and inflammation in myocardial injury in diabetic rats. The underlying molecular mechanism responsible for the cardio-protection role of morin, might be the cross-talk between MAPK/Akt/GSK3β, Nrf2/HO-1, and inflammasome pathways.

### Supplementary Information

**Additional file 1**. Representative Western blot images with respective loading control (GAPDH).

## Data Availability

The raw data of this study will be available by the First and corresponding authors only on valid requests.

## Abbreviations

AGE: Advance Glycation End products
BSA: Bovine Serum Albumin
HO-1: Heme Oxygenase-1
IL-6: Interleukin-6
ISO: Isoproterenol
MAPK: Mitogen Activated Protein Kinase
Nrf-2: Nuclear factor erythroid 2- related factor
PARP: Poly adenosine diphosphate-ribose polymerase
ROS: Reactive Oxygen Species
RAGE: Receptor of Advance Glycation End products

## References

[1] Saeedi P, Petersohn I, Salpea P, Malanda B, Karuranga S, Unwin N (2019). Global and regional diabetes prevalence estimates for 2019 and projections for 2030 and 2045: results from the International Diabetes Federation Diabetes Atlas. Diabetes Res Clin Pract.

[2] Lee YB, Han K, Kim B, Lee SE, Jun JE, Ahn J (2019). Risk of early mortality and cardiovascular disease in type 1 diabetes: a comparison with type 2 diabetes, a nationwide study. Cardiovasc Diabetol.

[3] Daffu G, del Pozo CH, O'Shea KM, Ananthakrishnan R, Ramasamy R, Schmidt AM (2013). Radical roles for RAGE in the pathogenesis of oxidative stress in cardiovascular diseases and beyond. Int J Mol Sci.

[4] David JA, Rifkin WJ, Rabbani PS, Ceradini DJ (2017). The Nrf2/Keap1/ARE Pathway and oxidative stress as a therapeutic target in type II diabetes mellitus. J Diabetes Res.

[5] Duan J, Guan Y, Mu F, Guo C, Zhang E, Yin Y (2017). Protective effect of butin against ischemia/reperfusion-induced myocardial injury in diabetic mice: involvement of the AMPK/GSK-3β/Nrf2 signaling pathway. Sci Rep.

[6] Zhang L, Guo Z, Wang Y, Geng J, Han S (2019). The protective effect of kaempferol on heart via the regulation of Nrf2, NF-κβ, and PI3K/Akt/GSK-3β signaling pathways in isoproterenol-induced heart failure in diabetic rats. Drug Dev Res.

[7] An S, Wang X, Shi H, Zhang X, Meng H, Li W (2020). Apelin protects against ischemia-reperfusion injury in diabetic myocardium via inhibiting apoptosis and oxidative stress through PI3K and p38-MAPK signaling pathways. Aging (Albany NY).

[8] Khamchai S, Chumboatong W, Hata J, Tocharus C, Suksamrarn A, Tocharus J (2020). Morin protects the blood-brain barrier integrity against cerebral ischemia reperfusion through anti-inflammatory actions in rats. Sci Rep.

[9] Kuzu M, Kandemir FM, Yildirim S, Kucukler S, Caglayan C, Turk E (2018). Morin attenuates doxorubicin-induced heart and brain damage by reducing oxidative stress, inflammation and apoptosis. Biomed Pharmacother.

[10] Ozdemir S, Kucukler S, Çomaklı S, Kandemir FM (2022). The protective effect of Morin against ifosfamide-induced acute liver injury in rats associated with the inhibition of DNA damage and apoptosis. Drug Chem Toxicol.

[11] Singh MP, Sharma C, Kang SC (2021). Morin hydrate attenuates adenine-induced renal fibrosis via targeting cathepsin D signaling. Int Immunopharmacol.

[12] Xu M, Zhang Y (2019). Morin inhibits ovarian cancer growth through the inhibition of NF-κB signaling pathway. Anticancer Agents Med Chem.

[13] Verma VK, Malik S, Narayanan SP, Mutneja E, Sahu AK, Bhatia J (2019). Role of MAPK/NF-κB pathway in cardioprotective effect of Morin in isoproterenol induced myocardial injury in rats. Mol Biol Rep.

[14] Paoli P, Cirri P, Caselli A, Ranaldi F, Bruschi G, Santi A (2013). The insulin-mimetic effect of Morin: a promising molecule in diabetes treatment. Biochim Biophys Acta.

[15] Razavi T, Kouhsari SM, Abnous K (2019). Morin exerts anti-diabetic effects in human HepG2 cells via down-regulation of miR-29a. Exp Clin Endocrinol Diabetes.

[16] Ohkawa H, Ohishi N, Yagi K (1979). Assay for lipid peroxides in animal tissues by thiobarbituric acid reaction. Anal Biochem.

[17] Moron MS, Depierre JW, Mannervik B (1979). Levels of glutathione, glutathione reductase and glutathione S-transferase activities in rat lung and liver. Biochim Biophys Acta.

[18] Bradford MM (1976). A rapid and sensitive method for the quantitation of microgram quantities of protein utilizing the principle of protein-dye binding. Anal Biochem.

[19] Marklund S, Marklund G (1974). Involvement of the superoxide anion radical in the autoxidation of pyrogallol and a convenient assay for superoxide dismutase. Eur J Biochem.

[20] Szkudelski T (2012). Streptozotocin-nicotinamide-induced diabetes in the rat Characteristics of the experimental model. Exp Biol Med (Maywood).

[21] Cheng D, Liang B, Li Y (2013). Antihyperglycemic effect of Ginkgo biloba extract in streptozotocin-induced diabetes in rats. Biomed Res Int.

[22] Suchal K, Malik S, Khan SI, Malhotra RK, Goyal SN, Bhatia J (2017). Protective effect of mangiferin on myocardial ischemia-reperfusion injury in streptozotocin-induced diabetic rats: role of AGE-RAGE/MAPK pathways. Sci Rep.

[23] Thakur V, Alcoreza N, Delgado M, Joddar B, Chattopadhyay M (2021). Cardioprotective effect of glycyrrhizin on myocardial remodeling in diabetic rats. Biomolecules.

[24] Sang L, Wang XM, Xu DY, Sang LX, Han Y, Jiang LY (2017). Morin enhances hepatic Nrf2 expression in a liver fibrosis rat model. World J Gastroenterol.

[25] Elmore S (2007). Apoptosis: a review of programmed cell death. Toxicol Pathol.

[26] Bratton SB, Salvesen GS (2010). Regulation of the Apaf-1–caspase-9 apoptosome. J Cell Sci.

[27] Paul C, Manero F, Gonin S, Kretz-Remy C, Virot S, Arrigo AP (2002). Hsp27 as a negative regulator of cytochrome C release. Mol Cell Biol.

[28] Chen Y, Li Y, Xu H, Li G, Ma Y, Pang YJ (2017). Morin mitigates oxidativestress, apoptosis and inflammation in cerebral ischemic rats. Afr J Tradit Complement Altern Med.

[29] Liu S, Wu N, Miao J, Huang Z, Li X, Jia P (2018). Protective effect of morin on myocardial ischemia-reperfusion injury in rats. Int J Mol Med.

[30] Li H, Song F, Duan LR, Sheng JJ, Xie YH, Yang Q (2016). Paeonol and danshensu combination attenuates apoptosis in myocardial infarcted rats by inhibiting oxidative stress: Roles of Nrf2/HO-1 and PI3K/Akt pathway. Sci Rep.

[31] Rizvi F, Mathur A, Kakkar P (2015). Morin mitigates acetaminophen-induced liver injury by potentiating Nrf2 regulated survival mechanism through molecular intervention in PHLPP2-Akt-Gsk3β axis. Apoptosis.

[32] Wu B, Huang XY, Li L, Fan XH, Li PC, Huang CQ (2019). Attenuation of diabetic cardiomyopathy by relying on kirenol to suppress inflammation in a diabetic rat model. J Cell Mol Med.

[33] Zhang H, Lu X, Liu Z, Du K (2018). Rosuvastatin reduces the pro-inflammatory effects of adriamycin on the expression of HMGB1 and RAGE in rats. Int J Mol Med.

[34] Guo H, Callaway JB, Ting JP (2015). Inflammasomes: mechanism of action, role in disease, and therapeutics. Nat Med.

[35] Takahashi M (2019). Role of NLRP3 Inflammasome in Cardiac Inflammation and Remodeling after Myocardial Infarction. Biol Pharm Bull.

[36] Li G, Wang G, Li M, Li L, Liu H, Sun M (2020). Morin inhibits Listeria monocytogenes virulence in vivo and in vitro by targeting listeriolysin O and inflammation. BMC Microbiol.

