# Supplementary material for: Morin ameliorates myocardial injury in diabetic rats via modulation of inflammatory pathways
Source: Lab Anim Res. 2024 Feb 9;40:3. doi: 10.1186/s42826-024-00190-x (PMC10854036; doi:10.1186/s42826-024-00190-x)
Supplement: Supplementary file 1 — Additional file 1. Representative Western blot images with respective loading control (GAPDH). [file 42826_2024_190_MOESM1_ESM.pptx]

## Slide 1
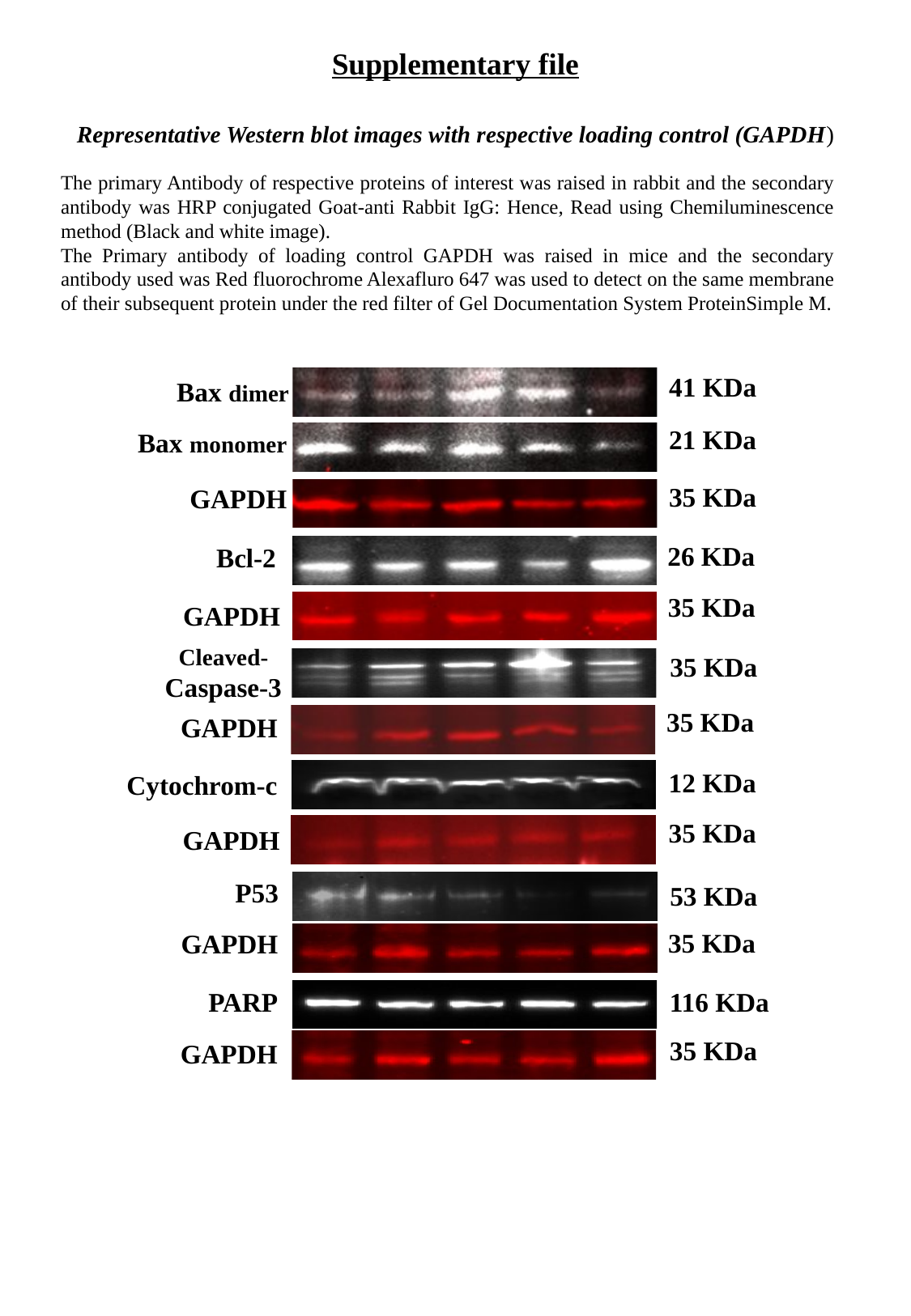

Supplementary file
Representative Western blot images with respective loading control (GAPDH)
The primary Antibody of respective proteins of interest was raised in rabbit and the secondary antibody was HRP conjugated Goat-anti Rabbit IgG: Hence, Read using Chemiluminescence method (Black and white image).
The Primary antibody of loading control GAPDH was raised in mice and the secondary antibody used was Red fluorochrome Alexafluro 647 was used to detect on the same membrane of their subsequent protein under the red filter of Gel Documentation System ProteinSimple M.
41 KDa
Bax dimer
Bax monomer
21 KDa
35 KDa
GAPDH
26 KDa
Bcl-2
GAPDH
35 KDa
Cleaved-
Caspase-3
GAPDH
35 KDa
35 KDa
12 KDa
Cytochrom-c
GAPDH
35 KDa
P53
GAPDH
53 KDa
35 KDa
116 KDa
PARP
35 KDa
GAPDH

## Slide 2
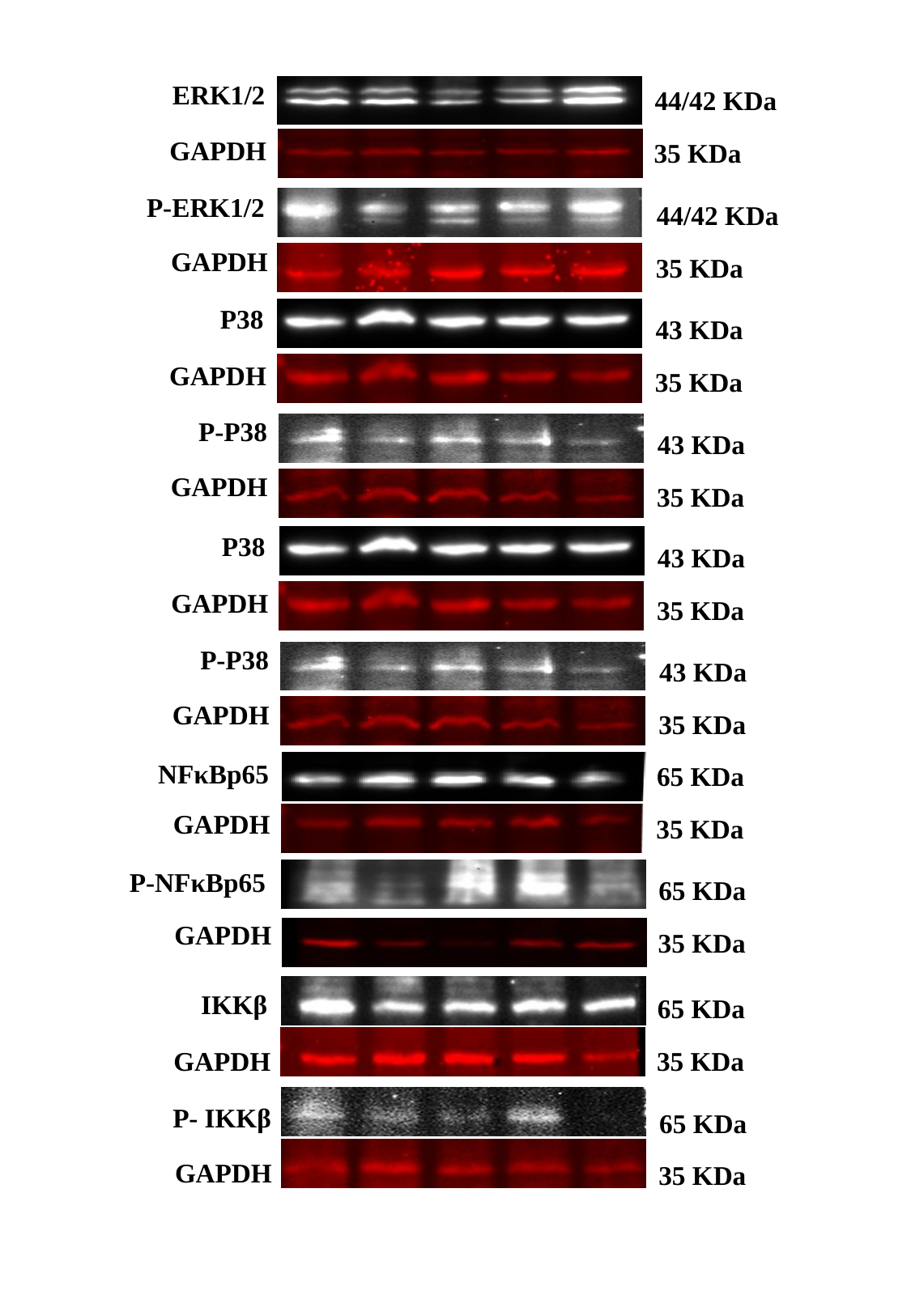

ERK1/2
44/42 KDa
GAPDH
35 KDa
P-ERK1/2
44/42 KDa
GAPDH
35 KDa
P38
43 KDa
GAPDH
35 KDa
P-P38
43 KDa
GAPDH
35 KDa
P38
43 KDa
GAPDH
35 KDa
P-P38
43 KDa
GAPDH
35 KDa
NFκBp65
65 KDa
GAPDH
35 KDa
P-NFκBp65
65 KDa
GAPDH
35 KDa
IKKβ
65 KDa
35 KDa
GAPDH
P- IKKβ
65 KDa
GAPDH
35 KDa

## Slide 3
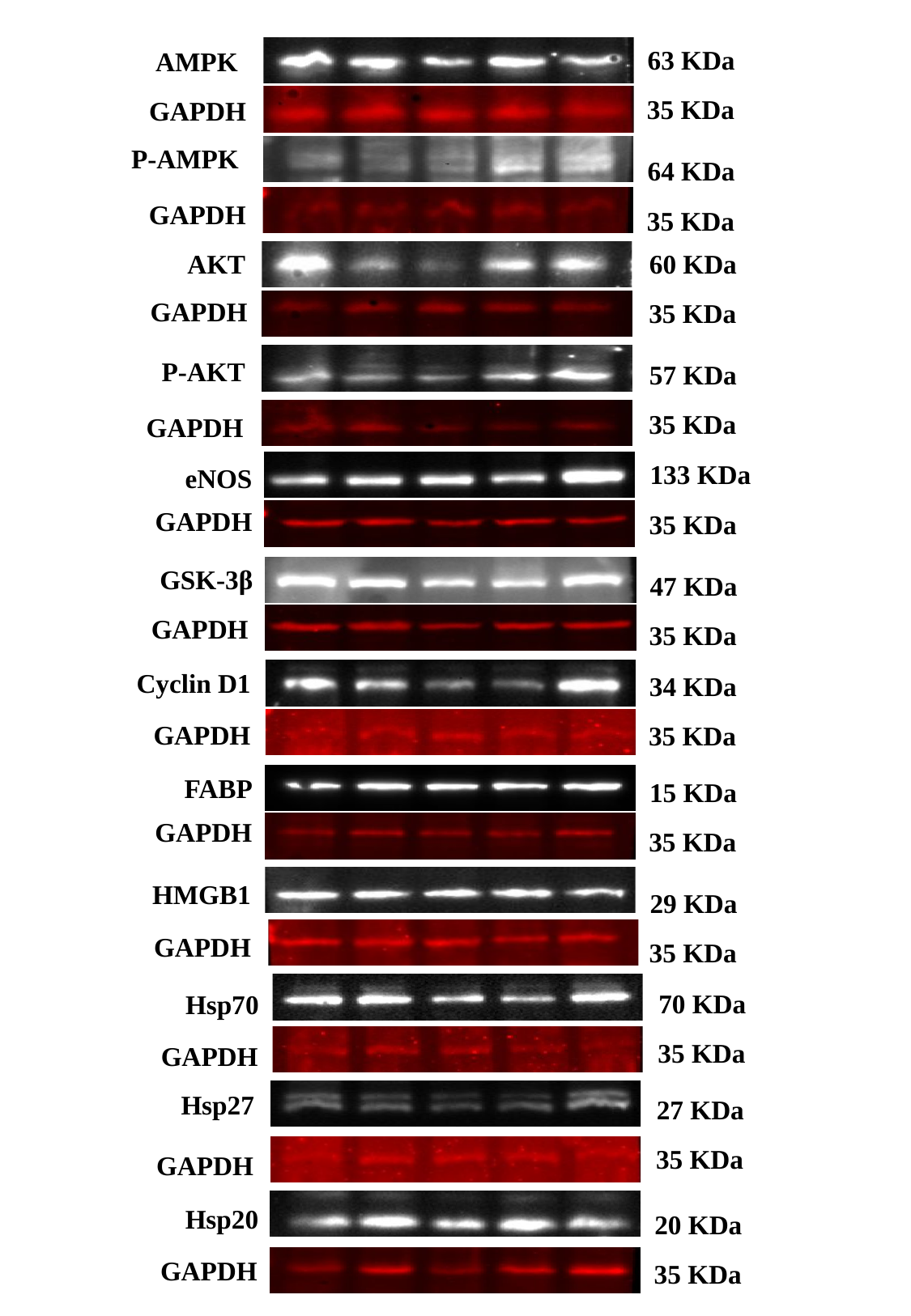

63 KDa
AMPK
35 KDa
GAPDH
P-AMPK
64 KDa
GAPDH
35 KDa
AKT
60 KDa
GAPDH
35 KDa
P-AKT
57 KDa
35 KDa
GAPDH
133 KDa
eNOS
GAPDH
35 KDa
GSK-3β
47 KDa
GAPDH
35 KDa
Cyclin D1
34 KDa
GAPDH
35 KDa
FABP
15 KDa
GAPDH
35 KDa
HMGB1
29 KDa
GAPDH
35 KDa
70 KDa
Hsp70
35 KDa
GAPDH
Hsp27
27 KDa
35 KDa
GAPDH
Hsp20
20 KDa
GAPDH
35 KDa

## Slide 4
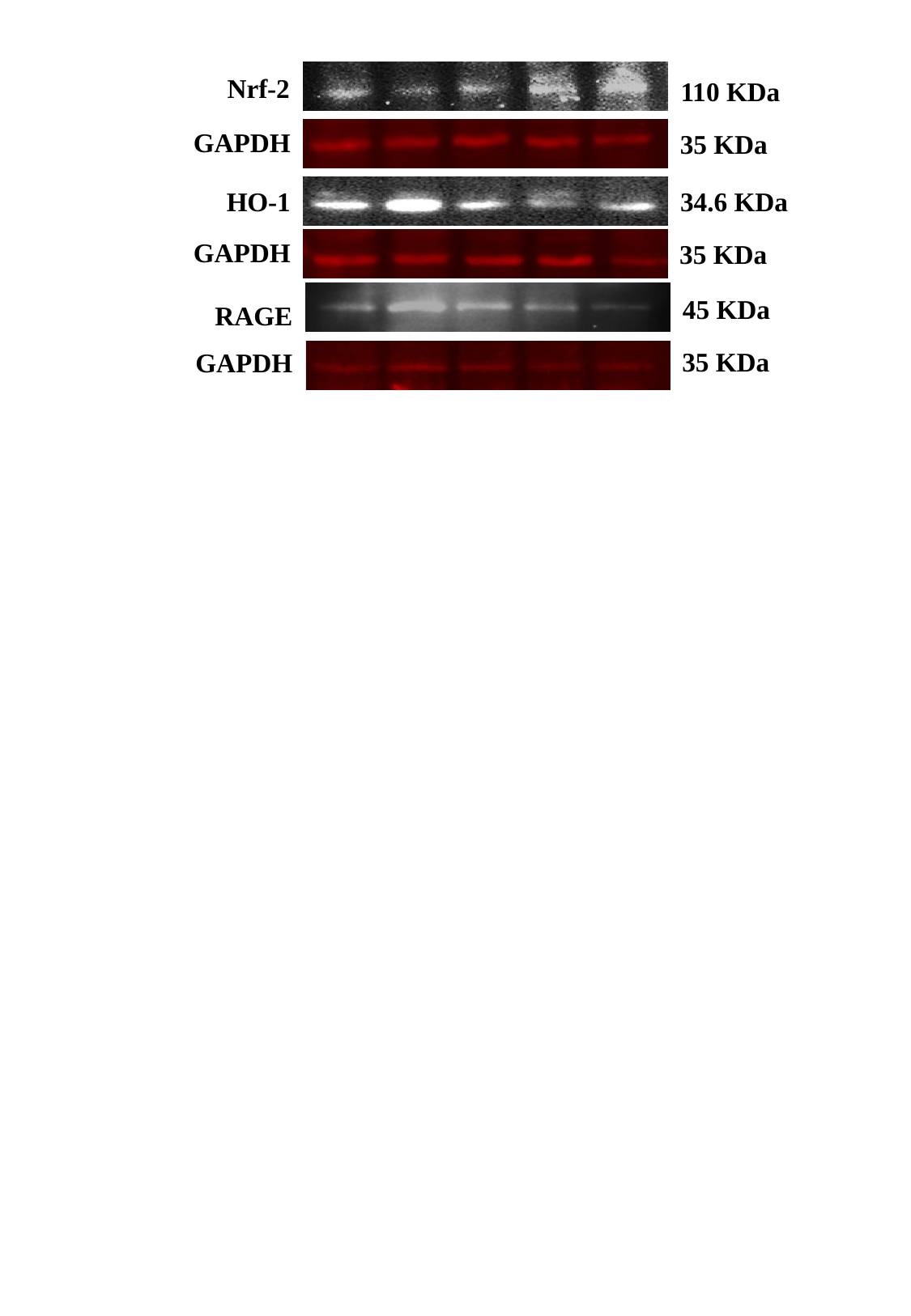

Nrf-2
110 KDa
GAPDH
35 KDa
HO-1
34.6 KDa
GAPDH
35 KDa
45 KDa
RAGE
35 KDa
GAPDH
